# Forced vital capacity and body mass index of Xinjiang children and adolescents: an analysis based on seven successive national surveys, 1985–2014

**DOI:** 10.1186/s12889-024-19072-x

**Published:** 2024-06-07

**Authors:** Feng Zhang, Cunjian Bi, Xiaojian Yin, Yuan Liu, Yaru Guo, Pengwei Sun, Jun Hong, Yanyan Hu

**Affiliations:** 1 Key Laboratory of Adolescent Health Assessment and Exercise Intervention of Ministry of Education, East China Normal University, Shanghai, 200241 China; 2 College of Physical Education and Health, East China Normal University, Shanghai, 200241 China; 3 School of Physical Education, Chizhou University, Chizhou, 247000 China; 4 College of Economics and Management, Shanghai Institute of Technology, Shanghai, 201418 China; 5 Research Department of Physical Education, Xinjiang Institute of Engineering, Urumqi, 830023 China; 6 College of Physical Education, Shanghai University, Shanghai, China

**Keywords:** FVC, Obesity, Xinjiang children and adolescents, Inversed U-shape

## Abstract

**Background:**

Pulmonary function is very important for the healthy development of children and adolescents. However, fewer studies have been conducted on pulmonary function trends in children and adolescents in remote areas. The aim of this study was to estimate the forced vital capacity (FVC) trend and its relationship with body mass index (BMI) among young people in Xinjiang during 1985–2014 using data from seven successive national surveys.

**Methods:**

A total of 19,449 Xinjiang children and adolescents aged 7–18 years were extracted from the Chinese National Survey on Students’ Constitution and Health. Height, weight, and FVC were measured repeatedly in each survey. FVC comparisons between adjacent surveys by age and sex were conducted by nonparametric Kruskal-Wallis after Kolmogorov-Smirnov of normality. One-way ANOVA and least significant difference(LSD) method was used to compare differences in FVC levels of Xinjiang children and adolescents with different BMI. The relationship between BMI and FVC was investigated using a nonlinear regression model.

**Results:**

The FVC levels of Xinjiang children and adolescents peaked in 2000, with overall FVC levels being 8.7% higher in 2000 than in 1985. Since then, a substantial decline occurred, contrasting to 2000, with FVC levels decreasing by 27% in 2014, which was still lower than that in 1985 by 20.73%. The proportion of overnutrition boys increased from 0.2% in 1985 to 22.1% in 2014, and girls from 0.5% in 1985 to 14.5% in 2014. An inverted U-shape association between FVC and BMI values was obtained for Xinjiang children and adolescents.

**Conclusions:**

Targeted measures should be carried out in schools to control BMI levels to ensure good lung function in children and adolescents in Xinjiang. Future studies should pay more attention to other factors affecting FVC, such as dietary behaviour, physical activity, and racial differences among children and adolescents.

## Introduction

As one of the significant parameters of spirometry, which was designed by Hutchinson more than one and a half centuries ago to determine the ‘capacity for life,’ the FVC is known as a common indicator of lung function, measuring the total volume exhaled after a maximum inspiration and has been widely used on diseases diagnosis and prediction [1]. For example, it can be used as the golden approach for the diagnosis of Chronic Obstructive Pulmonary Disease in clinical practices [2]. Studies also showed that, FVC has an important role in the diagnosis of interstitial lung disease (ILD) [3] and the assessment of early respiratory infections in children [4]. In addition, FVC is commonly used as an indicator in the assessment of child and adolescent population lung health and the effectiveness of related exercise interventions [5, 6].

The associations of obesity with reduced FVC have received considerable attention [7–9] Obesity, evaluated by BMI, is inversely associated with lung function both in a Mediterranean and Caribbean population [10–12]. A meta-analysis also suggested physicians should be aware of the adverse effects of obesity on lung function, and weight control should be considered in the management of airway disease among the obese [13]. However, to our best knowledge, the relationship between BMI and FVC in China was rarely studied, not to mention the outlying districts of west China—the Xinjiang Uygur Autonomous Region.

Located in north-western China, Xinjiang has a temperate continental climate with a lower average temperature compared with other provinces [14]. A recent study found that low ambient temperature was significantly associated with a decrease of FVC in Chinese school children [15]. We have reported that the average BMI-for-age z-score of children and adolescents in Xinjiang increased since 1985 [16].

Therefore, the FVC situation of Xinjiang school children needs to be worried about. The present study aimed to estimate the FVC trend and its relationship with BMI among young people in Xinjiang during 1985–2014 using data from seven successive national surveys.

## Materials and methods

### Participants and sampling

Data were drawn from the Chinese National Survey on Students’ Constitution and Health (CNSSCH) in 1985–2014. CNSSCH is jointly conducted by six national departments every four/five years since 1985 and has been conducted seven times by 2014 in 31 provinces throughout China. We draw data of Xinjiang children and adolescents aged 7–18 years old from the seven times CNSSCH survey.

Considering this socioeconomic context and the national distribution, children and adolescents without mental and physically disability from Urumqi, Yining and Akesu were selected using multistage stratified cluster sampling in Xinjiang since 1985. Kashgar, Kizilsu Kirgiz Autonomous Prefecture and Altay Prefecture were also included after 2010. In this study, 2880, 2880, 2880, 2400, 2880, 2880 and 2880 people were tested in 1985, 1990, 1995, 2000, 2005, 2010 and 2014 respectively. A total of 19,680 people. A total of 231missing data or biologically implausible values were excluded from study (*n* = 22, 0.76% in 1985; *n* = 12, 0.42% in 1990; *n* = 29, 1% in 1995; *n* = 33, 1.37% in 2000; *n* = 59, 2.05% in 2005; *n* = 44, 1.55% in 2010; *n* = 32, 1.12% in 2014). The 231 individuals excluded from this study had an age distribution of 7–18 years. The reasons for exclusion were mainly due to the fact that important demographic information was not filled in completely, such as age and gender information was not filled in and therefore could not be analysed and participants were excluded. Another reason, there were significant extreme values in the already filled in values of height or weight assessment such as height of 40 cm and hence were excluded in this study. Finally, 19449 participants(*N* = 2858 in 1985; *N* = 2868 in 1990; *N* = 2851 in 1995; *N* = 2367 in 2000; *N* = 2821 in 2005; *N* = 2836 in 2010; *N* = 2848 in 2014) were obtained for the present study. The effective data response rates were 99.24%, 99.58%, 98.99%, 98.63%, 97.95%, 98.47%, 98.89%. There were no significant differences in sex and socio-economic status of the participants.

### Anthropometric measurements

Before the investigation, all the testers underwent strict training and qualified for CNSSCH test. Each project was tested by fixed specially-assigned personnel. Calibration of the instrument was carried out before the test every day. demographic information including age, sex, grade, the city was collected and height, weight, FVC were tested according to the CNSSCH test guidelines [17].

Students were required to stand on the altimeter with their shoes and socks being taken off during the height test. The result was recorded to the nearest 0.1 cm by use of the mean of three measurements. Weight was tested by lever scale before 2000, and electronic scales were used after 2005. Students were required to wear only light clothing and stand erect, barefoot, and at ease while being measured. The result was recorded to the nearest 0.1 kg by use of the mean of three measurements. During measuring FVC, a rotary spirometer(FHL-2 Rotary Spirometer) was used before 2000. With the development of measuring instrument technology, an electronic spirometer(FHL-001 Electronic Spirometer) was adopted in 2005, 2010, and 2014. Before the test, the test method was introduced by trained staff. The test is carried out twice, and the one with the best score was recorded as the result. The result was recorded to the nearest 0.1 ml.

### Evaluation criteria for nutrition

BMI was calculated by weight over height squared (kg/m²). Z scores for BMI and height were calculated as an individual’s specific result minus the median values and then divided by the standard deviation (SD) for this individual’s age and sex in the WHO reference population [18]. According to the WHO standards and classifications, the growth and BMI were divided as stunting (<–2 for height Z score), thinness (<–2 for BMI Z score), normal (≥–2 and ≤ 1 for BMI Z score, and ≥–2 for height Z score), overweight (> 1 for BMI Z score) and obesity (> 2 for BMI Z score). Additionally, both stunting and thinness were combined as undernutrition, and both overweight and obesity were combined as overnutrition [19].

### Ethical consideration

This study was conducted in accordance with the Declaration of Helsinki and approved by human Subject Protection Committee of Chizhou University (24574111). The written informed consent was obtained from all the students and their parents before the investigation. and the test was conducted after the paper informed consent was signed. The test was conducted by questionnaire coding to protect students’ privacy.

### Statistical analysis

Descriptive statistics were used to describe the mean and SD of height, weight, BMI, and FVC for Xinjiang children and adolescents for each survey year from 1985 to 2014, and the proportions of undernutrition, normal, and overnutrition were also provided. Taking the mean and SD of FVC in 1985 as a reference, FVC values in 1991, 1995, 2000, 2005, 2010, and 2014 were standardized and comparisons between adjacent surveys by age and sex were conducted by nonparametric Kruskal-Wallis after Kolmogorov-Smirnov of normality. The following formula was used in this study to calculate these z-scores. For example, the calculation of z-scores for 7-year-old boys in 2014: 2014 FVC-z-scores = (2014 FVC actual test value − 1985 FVC reference mean)/1985 FVC reference standard deviation. Because FVC-z-scores are non-normally distributed data, P50 (P25, P75) to present the results. One-way ANOVA and least significant difference(LSD) method were used to compare differences in FVC levels of Xinjiang children and adolescents with different BMI (undernutrition, normal, overnutrition). The relationship between BMI and FVC was investigated using a nonlinear regression model: Y(FVC) = a + bBMI + cBMI^2^(where a, b, and c were all constants). FVC was considered as the dependent variable, and BMI as the independent variable. The level of statistical significance was set at 0.05. All analyses were conducted using IBM SPSS version 25.0 (IBM Corp., Armonk, NY, USA) and GraphPad Prism 8.0.2(GraphPad Software, Inc., CA, USA).

## Results

In 1985, 1991, 1995, 2000, 2005, 2010 and 2014, there was no significant difference between the average age of boys and girls and the ratio of the number of boys to the number of girls. Table 1 shows the height, weight, BMI and FVC in Xinjiang children and adolescents aged 7–18 years old in the CNSSCH, from 1985 to 2014. The overall results showed that FVC levels among children and adolescents in Xinjiang, China, showed an increasing trend between 1985 and 2000, followed by a decreasing trend until 2014.

**Table 1:** Height, weight, BMI and FVC in Xinjiang children and adolescents aged 7–18 years old in the CNSSCH, from 1985 to 2014.[Mean (SD)]

	1985	1991	1995	2000	2005	2010	2014
**Boys(7-18y)**							
Sample size(%)	1430(50.03)	1435(50.03)	1427(50.05)	1187(50.15)	1411(50.02)	1419(50.04)	1423(49.96)
Age (years)	12.50(3.45)	12.49(3.45)	12.50(3.45)	12.50(3.45)	12.51(3.45)	12.50(3.45)	12.49(3.45)
Height (cm)	147.32(18.05)	150.88(17.45)	150.47(18.54)	152.4(18.82)	151.67(18.72)	150.58(17.90)	151.19(18.53)
Weight (kg)	37.56(13.34)	40.22(13.70)	40.43(14.18)	42.61(15.02)	43.16(15.55)	42.44(14.50)	44.88(15.88)
BMI (kg/m^2^)	16.62(2.27)	17.03(2.53)	17.16(2.49)	17.67(2.84)	18.05(3.09)	18.09(2.87)	18.94(3.21)
Undernutrition(n,%)	188(13.1)	86(6.0)	113(7.9)	56(4.7)	72(5.1)	120(8.5)	69(4.8)
Normal(n,%)	1239(86.6)	1302(90.7)	1265(88.6)	999(84.2)	1145(81.1)	1086(76.5)	1039(73.0)
Overnutrition(n,%)	3(0.2)	47(3.3)	49(3.4)	132(11.1)	194(13.7)	213(15.0)	315(22.1)
FVC (ml)	2539.74(1031.84)	2688.89(1028.49)	2742.19(1083.19)	2799.89(1076.46)	2047.02(941.24)	2215.84(916.01)	2116.45(991.05)
**Girls(7-18y)**							
Sample size(%)	1428(49.97)	14,338(49.97)	1424(49.95)	1180(49.85)	1410(49.98)	1417(49.96)	1425(50.04)
Age (years)	12.49(3.45)	12.50(3.45)	12.50(3.45)	12.49(3.45)	12.50(3.45)	12.48(3.45)	12.51(3.45)
Height (cm)	144.69(15.07)	147.28(13.80)	146.45(14.73)	147.79(14.89)	147.22(14.8)	146.06(14.56)	146.39(14.99)
Weight (kg)	36.32(11.87)	37.89(11.33)	37.67(11.78)	38.96(11.99)	39.48(12.28)	39.54(12.39)	41.23(12.97)
BMI (kg/m^2^)	16.76(2.66)	16.99(2.68)	17.03(2.73)	17.32(2.73)	17.7(2.91)	18(3.05)	18.67(3.15)
Undernutrition(n,%)	155(10.9)	56(3.9)	106(7.4)	55(4.7)	71(5.0)	125(8.8)	106(7.4)
Normal(n,%)	1266(88.7)	1348(94.1)	1298(91.2)	1077(91.3)	1226(87.0)	1149(81.1)	1112(78.0)
Overnutrition(n,%)	7(0.5)	29(2.0)	20(1.4)	48(4.1)	113(8.0)	143(10.1)	207(14.5)
FVC (ml)	2121.37(656.29)	2189.67(623.68)	2172.4(674.38)	2264.54(682.65)	1509.63(554.97)	1702.04(556.53)	1579.26(599.74)
**Total(7-18y)**							
Sample size	2858	2868	2851	2367	2821	2836	2848
Age (years)	12.50(3.45)	12.50(3.45)	12.50(3.45)	12.50(3.45)	12.51(3.45)	12.49(3.45)	12.50(3.45)
Height (cm)	146.01(16.68)	149.08(15.83)	148.46(16.86)	150.1(17.13)	149.45(17.01)	148.32(16.47)	148.79(17.02)
Weight (kg)	36.94(12.64)	39.06(12.62)	39.05(13.11)	40.79(13.71)	41.32(14.13)	41(13.56)	43.06(14.61)
BMI (kg/m^2^)	16.69(2.48)	17.01(2.60)	17.1(2.62)	17.50(2.79)	17.87(3.01)	18.04(2.96)	18.8(3.19)
Undernutrition(n,%)	343(12.0)	142(5.0)	219(7.7)	111(4.7)	143(5.1)	245(8.6)	175(6.1)
Normal(n,%)	2505(87.6)	2650(92.4)	2563(89.9)	2076(87.7)	2371(84.0)	2235(78.8)	2151(75.5)
Overnutrition(n,%)	10(0.3)	76(2.6)	69(2.4)	180(7.6)	307(10.9)	356(12.6)	522(18.3)
FVC (ml)	2330.7(889.63)	2439.46(886.39)	2457.6(946.22)	2533.01(940.61)	1778.42(817.97)	1959.12(800.25)	1847.66(861.77)

Note: BMI: Body mass index; FVC: Forced vital capacity; CNSSCH: Chinese National Survey on Students’ Constitution and Health; SD: standard deviation

**Fig. 1:**
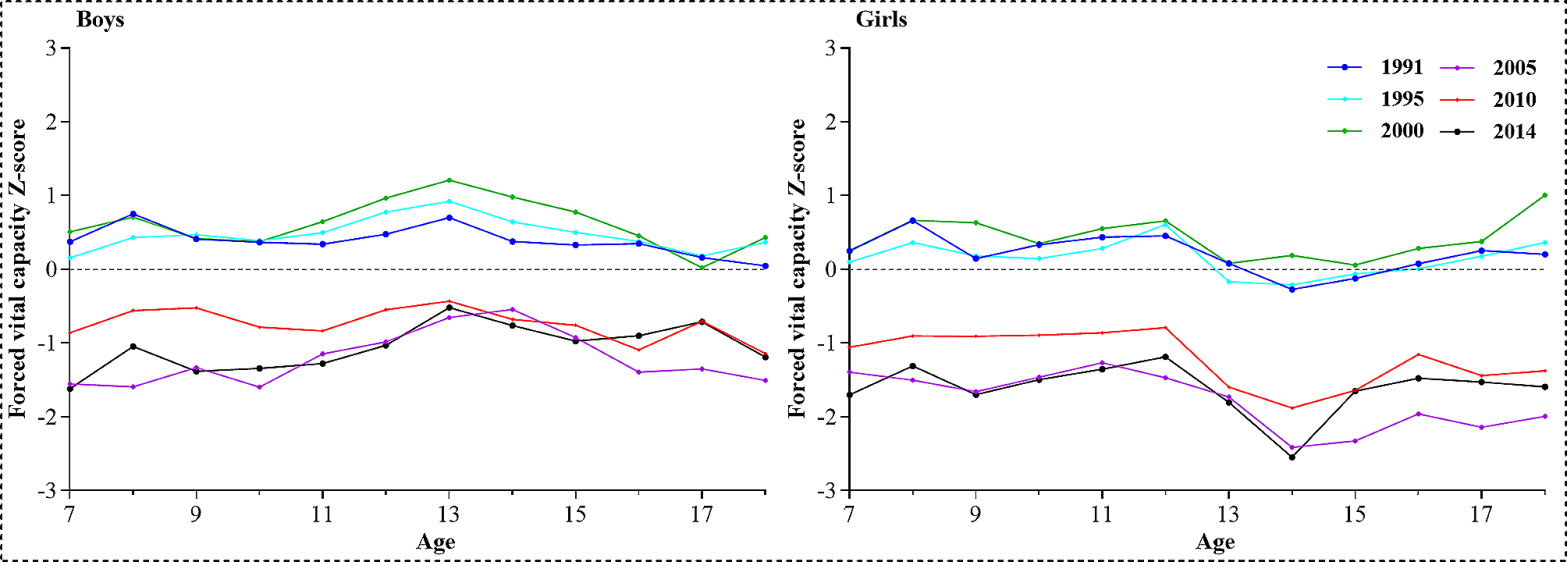
Variation trend of FVC -Z score in Xinjiang children and adolescents aged 7–18 years,1991–2014 (with 1985 as reference)

**Fig. 2:**
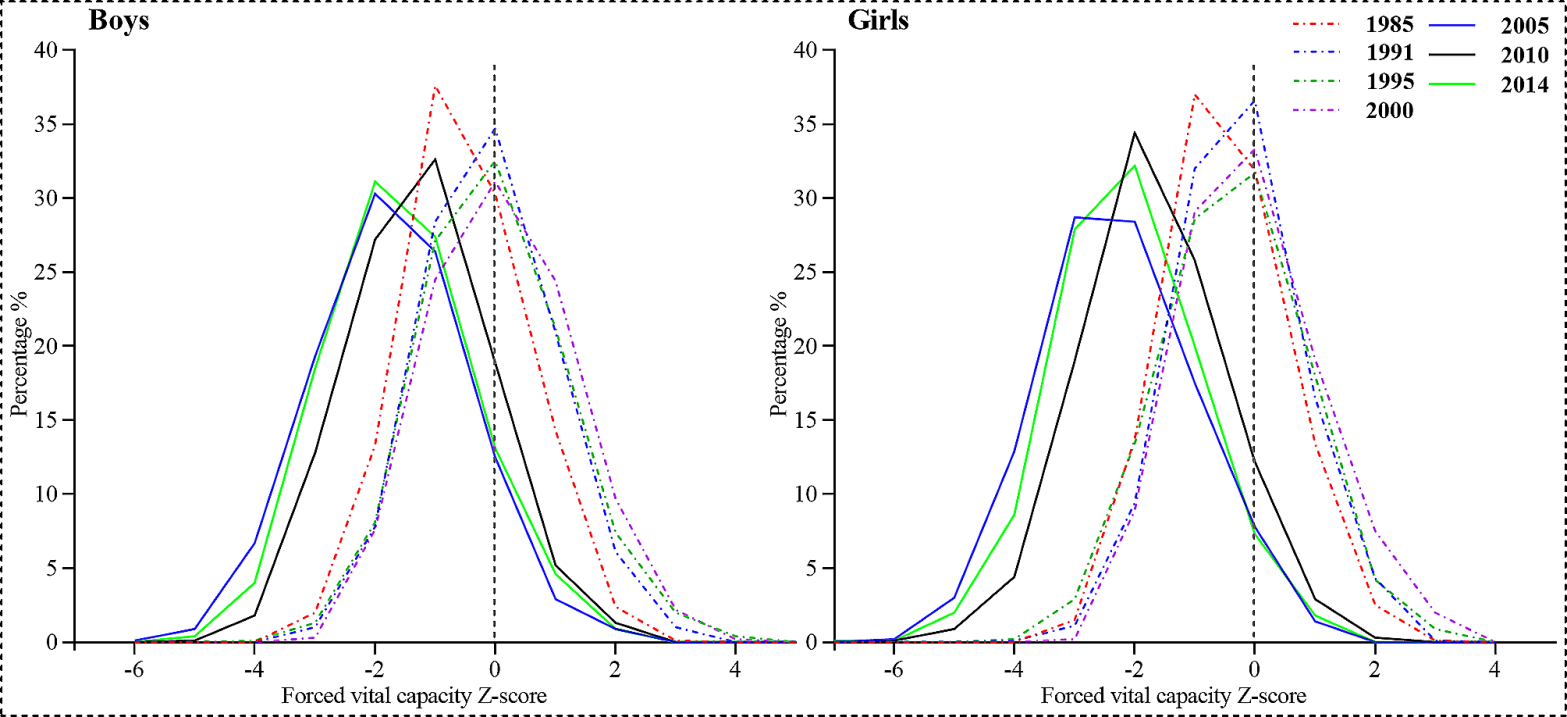
The FVC-for-age Z-score distribution curves for Xinjiang children and adolescents aged 7–18 years

Figure 1 shows that, for both boys and girls, the FVC-for-age z-score in 1991, 1995, and 2000 was generally higher than that in 1985; while after 2005, the FVC-for-age z-score was generally lower than that in 1985, showing a downward trend. Overall, The FVC levels of Xinjiang children and adolescents peaked in 2000, with overall FVC levels being 8.7% higher in 2000 than in 1985. Since then, a substantial decline occurred, contrasting to 2000, with FVC levels decreasing by 27% in 2014, which was still lower than that in 1985 by 20.73%. Figure 2 shows the FVC-for-age Z-score distribution curves for Xinjiang children and adolescents aged 7–18 years. As can be seen from the graph, the compared with 1985, the FVC distributions shifted to the right in 1991, 1995, and 2000 and shifted to the left in 2005, 2010, and 2014, It follows that the FVC level first increased and then decreased since 1985.

Taking the mean and SD of FVC in 1985 as a reference, FVC values in 1991, 1995, 2000, 2005, 2010, and 2014 were standardized and comparisons between adjacent surveys by age and sex were conducted by nonparametric Kruskal-Wallis after Kolmogorov-Smirnov of normality. Table 2 compared the FVC levels of Xinjiang children and adolescents by age and sex between adjacent surveys. The FVC levels of boys at 7–16 years old and girls at 8, 10–12, 17–18 years old in 1991 was significantly higher than that in 1985 (*P* < 0.05). There was no significant difference between 1991 and 1995 and between 1995 and 2000 in most age groups. While compared with 2000, the FVC levels in 2005 significantly decreased across all the age groups (*P* < 0.05). After 2005, the FVC levels significantly increased in most age groups (*P* < 0.05), but still lower than that in 1985.

**Table 2:** The comparison of the FVC-z-scores levels for Xinjiang children and adolescents by age and sex between adjacent surveys[P50 (P25, P75)]

	1985	1991	1995	2000	2005	2010	2014	Z^#^/*P*					
**Boys**								A	B	C	D	E	F
7	-0.05(-0.77, 0.59)	0.36(-0.46, 1.18)	0.03(-0.79, 1.10)	0.61(-0.28, 1.24)	-1.59(-2.37, -0.68)	-1.07(-1.85, 0.06)	-1.62(-2.45, -1.14)	**-2.561** ^**a**^	-1.453	-1.927	**-9.971** ^**b**^	**-4.025** ^**b**^	**-4.507** ^**b**^
8	-0.03(-0.70, 0.49)	0.59(-0.12, 1.55)	0.30(-0.53, 1.17)	0.72(-0.20, 1.57)	-1.59(-2.61, -0.68)	-0.64(-1.24, 0.19)	-1.10(-1.94, -0.27)	**-4.788** ^**b**^	**-2.156** ^**a**^	-1.868	**-9.950** ^**b**^	**-5.798** ^**b**^	**-3.098** ^**b**^
9	-0.03(-0.70, 0.83)	0.61(-0.46, 1.33)	0.26(-0.46, 1.33)	0.40(-0.61, 0.97)	-1.47(-2.11, -0.57)	-0.41(-1.46, 0.25)	-1.28(-2.35, -0.59)	**-2.973** ^**b**^	-0.190	-0.536	**-9.286** ^**b**^	**-5.406** ^**b**^	**-5.384** ^**b**^
10	0.11(-0.72, 0.73)	0.31(-0.43, 1.26)	0.55(-0.44, 1.19)	0.25(-0.65, 1.28)	-1.65(-2.49, -0.6)	-0.73(-1.86, 0.00)	-1.30(-2.14, -0.43)	**-2.490** ^**a**^	-0.238	-0.877	**-9.294** ^**b**^	**-4.579** ^**b**^	**-3.215** ^**b**^
11	-0.04(-0.66, 0.63)	0.57(-0.35, 1.00)	0.51(-0.35, 1.19)	0.70(-0.35, 1.59)	-1.03(-2.1, -0.26)	-0.77(-1.65, -0.03)	-1.40(-2.4, -0.38)	**-2.837** ^**a**^	-0.657	-1.116	**-8.279** ^**b**^	**-1.975** ^**a**^	-2.692
12	-0.13(-0.68, 0.69)	0.42(-0.35, 1.24)	0.69(-0.13, 1.79)	0.99(-0.06, 1.94)	-1.06(-1.74, -0.14)	-0.46(-1.28, 0.12)	-1.08(-1.88, -0.20)	**-3.256** ^**b**^	**-1.982** ^**a**^	-1.249	**-9.150** ^**b**^	**-2.794** ^**a**^	**-3.099** ^**b**^
13	-0.24(-0.66, 0.55)	0.59(-0.04, 1.22)	0.92(0.14, 1.64)	1.14(0.45, 1.94)	-0.78(-1.71, 0.25)	-0.47(-1.22, 0.26)	-0.67(-1.36, 0.20)	**-5.163** ^**b**^	-1.571	-1.728	**-9.452** ^**b**^	-1.548	-0.837
14	-0.11(-0.76, 0.81)	0.35(-0.30, 1.04)	0.77(-0.25, 1.61)	0.85(0.08, 1.73)	-0.54(-1.41, 0.16)	-0.76(-1.45, 0.15)	-0.86(-1.60, 0.07)	**-2.877** ^**b**^	-1.914	-1.811	**-8.459** ^**b**^	-0.632	-0.690
15	-0.01(-0.68, 0.58)	0.23(-0.51, 0.99)	0.49(-0.17, 1.17)	0.76(0.16, 1.36)	-0.97(-1.63, -0.24)	-0.80(-1.60, 0.01)	-1.05(-1.91, -0.17)	**-2.187** ^**a**^	-1.615	**-2.124** ^**a**^	**-10.258** ^**b**^	-1.261	-1.743
16	-0.03(-0.62, 0.67)	0.33(-0.24, 0.91)	0.33(-0.43, 1.10)	0.43(-0.16, 1.19)	-1.45(-2.35, -0.39)	-0.97(-1.88, -0.29)	-0.93(-1.86, -0.17)	**-2.691** ^**a**^	-0.031	-0.837	**-9.295** ^**b**^	-1.699	-0.866
17	0.01(-0.65, 0.69)	0.14(-0.48, 0.63)	0.16(-0.44, 0.74)	-0.05(-0.57, 0.54)	-1.37(-2.07, -0.6)	-0.73(-1.26, -0.18)	-0.75(-1.31, -0.08)	-1.110	-0.239	-1.604	**-8.851** ^**b**^	**-4.661** ^**b**^	-0.056
18	-0.02(-0.56, 0.71)	-0.02(-0.56, 0.71)	0.46(-0.35, 1.01)	0.22(-0.34, 1.24)	-1.60(-2.57, -0.59)	-1.24(-1.79, -0.58)	-1.22(-1.89, -0.43)	-0.272	**-2.626** ^**a**^	-0.380	**-9.098** ^**b**^	**-2.292** ^**a**^	-0.192
**Girls**													
7	0.03(-0.79, 0.57)	0.12(-0.43, 0.93)	0.03(-0.88, 0.93)	0.28(-1.06, 1.14)	-1.48(-2.46, -0.45)	-0.95(-2.12, -0.12)	-1.68(-2.58, -1.08)	-1.672	-1.134	-0.791	**-7.919** ^**b**^	**-2.152** ^**a**^	**-3.693** ^**b**^
8	-0.02(-0.84, 0.73)	0.73(-0.10, 1.37)	0.31(-0.51, 1.14)	0.56(-0.10, 1.30)	-1.62(-2.44, -0.65)	-1.09(-1.67, -0.06)	-1.48(-2.20, -0.55)	**-4.414** ^**b**^	-1.779	**-2.043** ^**a**^	**-10.319** ^**b**^	**-4.038** ^**b**^	**-2.886** ^**b**^
9	0.01(-0.72, 0.66)	0.01(-0.43, 0.75)	0.05(-0.72, 0.89)	0.53(-0.13, 1.33)	-1.67(-2.66, -0.82)	-1.09(-1.65, -0.16)	-1.87(-2.33, -1.12)	-1.300	-0.246	**-2.614** ^**a**^	**-10.760** ^**b**^	**-4.434** ^**b**^	**-5.762** ^**b**^
10	-0.05(-0.76, 0.62)	0.28(-0.44, 1.25)	0.20(-1.02, 0.92)	0.28(-0.5, 0.92)	-1.63(-2.32, -0.84)	-0.86(-1.84, -0.05)	-1.57(-2.30, -0.80)	**-2.622** ^**a**^	-1.411	-1.069	**-9.635** ^**b**^	**-3.903** ^**b**^	**-4.036** ^**b**^
11	-0.08(-0.78, 0.78)	0.29(-0.24, 1.10)	0.29(-0.51, 0.94)	0.45(-0.24, 1.20)	-1.29(-2.19, -0.31)	-0.94(-1.66, -0.04)	-1.38(-2.12, -0.74)	**-3.368** ^**b**^	-1.189	-1.527	**-9.287** ^**b**^	**-2.619** ^**a**^	**-3.391** ^**b**^
12	-0.02(-0.74, 0.67)	0.37(-0.19, 1.15)	0.63(-0.15, 1.35)	0.78(-0.15, 1.40)	-1.53(-2.15, -0.78)	-0.82(-1.53, -0.20)	-1.33(-1.96, -0.47)	**-3.461** ^**b**^	-1.381	-0.546	**-10.478** ^**b**^	**-4.552** ^**b**^	**-2.834** ^**b**^
13	-0.06(-0.58, 0.68)	-0.06(-0.37, 0.71)	-0.11(-0.83, 0.45)	0.20(-0.69, 0.71)	-1.85(-2.50, -0.90)	-1.64(-2.32, -0.90)	-1.86(-2.73, -1.10)	-0.658	-1.892	-1.846	**-9.519** ^**b**^	-1.052	-1.464
14	-0.07(-0.73, 0.47)	-0.20(-1.20, 0.47)	-0.20(-1.20, 0.80)	0.13(-0.67, 0.83)	-2.53(-3.50, -1.32)	-1.85(-3.14, -0.86)	-2.69(-3.66, -1.47)	-1.668	-0.239	**-2.223** ^**a**^	**-10.592** ^**b**^	**-2.597** ^**a**^	**-3.300** ^**b**^
15	-0.02(-0.71, 0.61)	-0.18(-0.86, 0.35)	-0.18(-0.86, 0.63)	-0.15(-0.64, 0.88)	-2.38(-3.24, -1.37)	-1.75(-2.27, -0.88)	-1.70(-2.52, -0.57)	-0.621	-0.304	-0.786	**-11.212** ^**b**^	**-4.345** ^**b**^	-0.324
16	-0.11(-0.70, 0.67)	0.03(-0.46, 0.52)	0.03(-0.70, 0.57)	0.18(-0.46, 0.67)	-2.06(-2.86, -1.02)	-1.25(-1.74, -0.59)	-1.35(-2.38, -0.72)	-0.932	-0.164	-1.168	**-10.861** ^**b**^	**-5.139** ^**b**^	**-2.102** ^**a**^
17	-0.10(-0.72, 0.76)	0.37(-0.34, 0.85)	0.37(-0.63, 1.09)	0.26(-0.44, 0.74)	-2.14(-2.86, -1.21)	-1.46(-2.14, -0.81)	-1.75(-2.56, -0.67)	**-1.977** ^**a**^	-0.108	-0.426	**-11.768** ^**b**^	**-4.881** ^**b**^	-1.237
18	-0.20(-0.77, 0.52)	0.24(-0.54, 0.77)	0.38(-0.51, 1.30)	0.67(0.24, 2.24)	-2.21(-2.94, -1.02)	-1.34(-1.89, -0.69)	-1.62(-2.36, -0.95)	**-1.977** ^**a**^	-1.226	**-3.436** ^**b**^	**-11.29** ^**b**^	**-4.22** ^**b**^	-1.794

Note: Taking the mean and SD of FVC in 1985 as a reference, FVC values in 1991, 1995, 2000, 2005, 2010, and 2014 were standardized and comparisons between adjacent surveys by age and sex were conducted by nonparametric Kruskal-Wallis after Kolmogorov-Smirnov of normality. The following formula was used in this study to calculate these z-scores. For example, the calculation of z-scores for 7-year-old boys in 2014: 2014 FVC-z-scores = (2014 FVC actual test value − 1985 FVC reference mean)/1985 FVC reference standard deviation. Because FVC-z-scores are non-normally distributed data, P50 (P25, P75) to present the results. ^**#**^ Mann-Whitney U test, derive the Z-value. A represent the comparison between 1985 and1991; B represent the comparison between 1991 and 1995; C represent the comparison between 1995 and 2000; D represent the comparison between 2000 and 2005; E represent the comparison between 2005 and 2010; F represent the comparison between 2010 and 2014. ^a^ represent *P* < 0.05, ^b^ represent *P* < 0.01

**Fig. 3:**
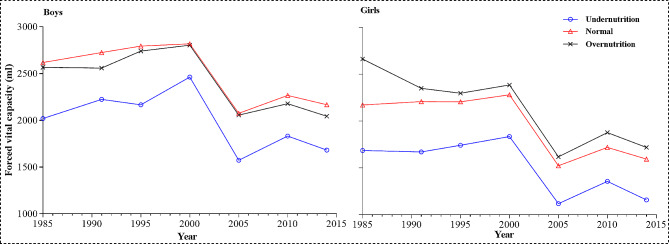
The FVC trends of children and adolescents aged 7–18 with different BMI in Xinjiang, China, 1985–2014

The result of one-way ANOVA shows that during 1985–2014, FVC levels of Xinjiang children and adolescents with different BMI are statistically significant except boys in the 2000 survey year (*P* < 0.001) (Table 3). Boys with normal BMI had the best FVC levels, followed by boys with overnutrition and undernutrition status. While girls with overnutrition status had the best FVC levels, followed by girls with normal and undernutrition status (Fig. 3). LSD pairwise comparison shows that children and adolescents with normal and undernutrition status were significantly different in all survey years (*P*<0.05), while children and adolescents with normal and overnutrition status were only significantly different in 1985, 2010, and 2014 survey years (Table 3).

**Table 3:** The FVC levels of Xinjiang children and adolescents with different nutrition statuses [Mean (SD)]

	Undernutrition	Normal	Overnutrition	I	II	III	F	*P* ^#^
**Boys**								
1985	2019.73(698.665)	2618.58(1051.032)	2566.67(1422.439)	598.85^a^	546.94	-51.91	28.551	<0.001
1991	2223.95(841.147)	2724.3(1035.044)	2558.72(954.681)	500.35^a^	334.77	-165.58	10.061	<0.001
1995	2165.66(833.889)	2793.77(1095.105)	2740.2(895.069)	628.11^a^	574.54^b^	-53.57	17.852	<0.001
2000	2461.61(1011.461)	2818.45(1087.596)	2802.95(999.133)	356.84^a^	341.34^b^	-15.50	2.924	0.054
2005	1572.71(641.256)	2075.37(953.558)	2055.7(917.934)	502.66^a^	482.99^b^	-19.67	9.790	<0.001
2010	1831.65(706.359)	2265.79(927.91)	2177.59(909.817)	434.14^a^	345.94^b^	-88.20	12.557	<0.001
2014	1682.28(779.758)	2167.44(991.527)	2043.35(1005.479)	485.16^a^	361.07^b^	-124.09	8.953	<0.001
**Girls**								
1985	1685.48(515.29)	2171.75(652.075)	2662.86(436.414)	486.27^a^	977.38^b^	491.11^c^	42.651	<0.001
1991	1670(384.386)	2207.82(621.363)	2349.66(668.551)	537.82^a^	679.66^b^	141.84	21.567	<0.001
1995	1740.75(586.846)	2205.71(671.332)	2298(510.558)	464.96^a^	557.25^b^	92.29	24.422	<0.001
2000	1834.55(598.869)	2281.08(684.47)	2386.25(560.655)	446.53^a^	551.7^b^	105.17	12.218	<0.001
2005	1117.35(378.423)	1522.41(553.496)	1617.5(568.532)	405.06^a^	500.15^b^	95.09	20.764	<0.001
2010	1355.27(468.45)	1718.01(548.741)	1876.76(569.082)	362.74^a^	521.49^b^	158.75^c^	33.233	<0.001
2014	1156.58(497.675)	1593.71(591.887)	1718.09(598.597)	437.13^a^	561.51^b^	124.38^c^	33.671	<0.001

Note: I represent the difference between normal weight group and undernutrition group, II represent the difference between undernutrition group and overnutrition group, III represent the difference between normal weight group and overnutrition group; ^a^ represent the comparison between normal weight group and undernutrition group (*P* < 0.05), ^b^ represent the comparison between undernutrition group and overnutrition group(*P* < 0.05), ^c^ represent the comparison between normal weight group and overnutrition group(*P* < 0.05). ^#^*P*-values are the comparative results of one-way ANOVA for FVC levels in children and adolescents with different nutritional status in Xinjiang

**Fig. 4:**
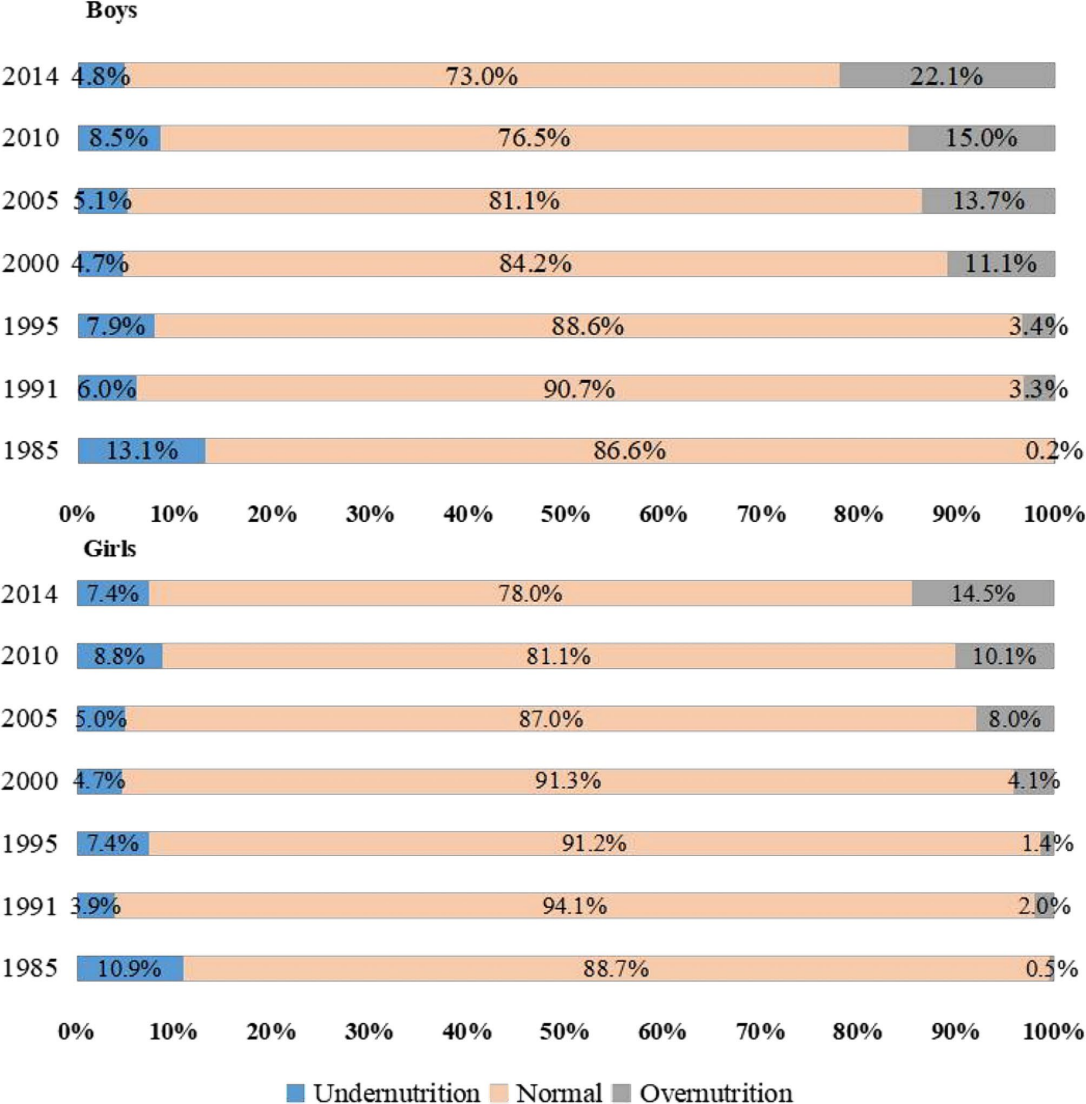
Distribution of BMI among children and adolescents in Xinjiang, China, 1985–2014

Figure 4 shows that the proportion of overnutrition students increased from 0.2% in 1985 to 22.1% in 2014 for boys, and from 0.5% in 1985 to 14.5% in 2014. The proportion of students with normal BMI, for both boys and girls, reached its peak in 1991 and then begin to decrease especially after 2000.

**Fig. 5:**
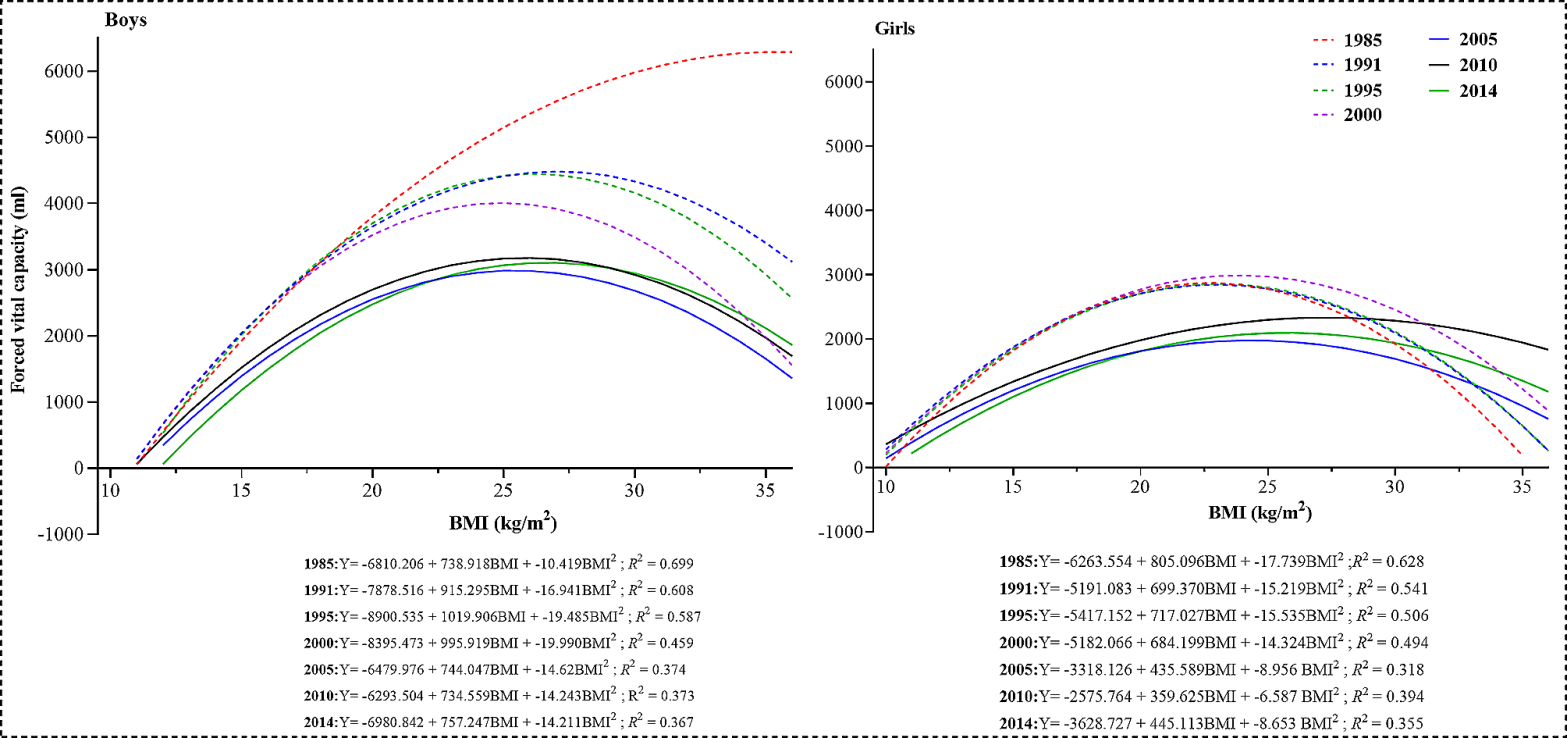
The association between FVC and BMI for Xinjiang children and adolescents, 1985–2014

An inverse U-shape association between FVC and BMI values was obtained for each survey in 1985–2014 except boys in 1985: both students with overnutrition and undernutrition had worse FVC levels than that of those with normal weight status (Fig. 5).

## Discussion

To the best of our knowledge, this longitudinal study for the first time focused on the FVC of Xinjiang children and adolescents from 1985 to 2014. The height, weight, BMI, and FVC of Xinjiang children and adolescents aged 7–18 years old all increased with age, especially in late adolescence, which was consistent with previous studies and the normal law of the healthy development of adolescents [20–22].

We observed that the FVC levels of Xinjiang children and adolescents peaked in 2000, with overall FVC levels being 8.7% higher in 2000 than in 1985. Since then, a substantial decline occurred, contrasting to 2000, with FVC levels decreasing by 27% in 2014, which was still lower than that in 1985 by 20.73%. Dong et al. found out that the FVC level of Chinese students peaked in 1995, they decreased sharply during 2005–2010, which was consistent with previous findings [23]. It has been reported that obesity-related parameters, such as BMI and WC, were significantly associated with low vital capacity after adjusting for confounding factors in both sexes [24]. A close relationship between obesity and restrictive pulmonary dysfunction has also been suggested [25, 26]. The declines in FVC correspond to the rise in the proportion of overnutrition children and adolescents in Xinjiang, which can partly explain the decline of FVC after 2000 in Xinjiang children and adolescents. Dong et al. suggested that students with stunting had the lowest FVC, and they explained that undernutrition reflected in high levels of stunting and thinness was the major contributor to the poor physical fitness of Chinese students, including FVC [20].

The 2018 Physical Activity Guidelines for Americans showed that the benefits of physical activity occur in generally healthy people of all ages, in people at risk of developing chronic diseases, and in people with chronic conditions or disabilities [27]. Low physical activity was reported to be independently associated with lung function decline in asthma patients [28, 29]. Evidence suggested that the average moderate-to-vigorous physical activity (MVPA) minutes per day among Chinese school-aged children is low, and less than one-third of them meet MVPA recommendations. Hence, the low physical activity level may be another reason for the decline of FVC in Xinjiang children and adolescents.

Studies have raised serious alarm over the threat of severe air pollution to lung development in Chinese school-age children [30, 31]. Gauderman et al. found out that long-term improvements in air quality were associated with statistically and clinically significant positive effects on lung-function growth in American children [32]. Animal experiments showed that the alveolar volume of mice exposed to high concentrations of PM2.5 decreased significantly, which may be a potential mechanism of air pollution affecting lung function in children and adolescents [33]. While the PM2.5 concentration increased slowly during 2000–2014, PM2.5 concentration exceeded the secondary limit (35 µg· m-3) in more than 65% of China [34]. These severe air pollution problems may partly explain the decline of FVC levels of Xinjiang children and adolescents.

We found an inverse U-shape association between FVC and BMI, with overnutrition and undernutrition students having worse FVC levels than normal-weight students. Studies have indicated that excessive accumulation of fat can change its mechanical action on the thoracic and abdominal cavity, resulting in changes in FVC and respiratory regulation, thus impairing lung function levels [35]. Similar inverse U-shapes were also found between BMI or waist circumference and cardiorespiratory fitness in Chinese children and adolescents [36]. It should be noted that the FVC levels in the undernutrition group were lower than those in the normal and overnutrition groups during 1985–2014. This result indicated the adverse effects of undernutrition on FVC in children and adolescents. With low body muscle mass, undernutrition children and adolescents are more likely to have less chest muscle strength, leading to decreased systolic ability and FVC levels.

### Strengths and limitations

The first advantage of the present study is the longitudinal design, which used seven surveys over 30 years. The second advantage of the present study lies in the large sample size, which was representative of Xinjiang children and adolescents. Nevertheless, we did not consider cofounders such as dietary behaviors, physical exercise, ethnic differences, which was the limitation of our study. Secondly, the use of different spirometers over time is another limitation that exists in this study. Third., in this study the FVC was tested twice, with the highest score being the outcome. However, it is common internationally to test three times, and the one with the highest test score was recorded as the result, which is also one of the limitations of this study.

## Conclusions

A representative sample of participants was included in this study, and it was proposed that BMI and FVC in children and adolescents in Xinjiang Province showed an inverted U-shaped curvilinear relationship. In view of this, targeted measures should be carried out in schools to control BMI levels to ensure good lung function in children and adolescents in Xinjiang. Meanwhile, future studies should pay more attention to other factors affecting FVC, such as dietary behaviour, physical activity, and racial differences among children and adolescents.

## Data Availability

The datasets used during the current study cannot be made publicly available as per ethics approval at Chizhou University. Readers can obtain them from the corresponding author on reasonable request.
